# Reversible Cerebral Vasoconstriction Syndrome Mimicking Eclampsia

**DOI:** 10.7759/cureus.57021

**Published:** 2024-03-27

**Authors:** Chalothorn Wannaphut, Yoshito Nishimura, Weiming Du, Chutawat Kookanok, Travis Watai, Christina Chong

**Affiliations:** 1 Internal Medicine, John A. Burns School of Medicine, University of Hawai'i, Honolulu, USA; 2 Internal Medicine, Phramongkutklao College of Medicine, Mahidol University, Bangkok, THA; 3 Internal Medicine, John A. Burns School of Medicine, University of Hawai’i, Honolulu, USA

**Keywords:** postpartum, eclampsia, call fleming syndrome, rcvs, reversible cerebral vasoconstriction syndrome

## Abstract

This report describes the case of an 18-year-old Micronesian pregnant woman at 32 weeks gestation, initially presumed to have eclampsia but later diagnosed with reversible cerebral vasoconstriction syndrome (RCVS). She presented with seizures, altered mental status, nystagmus, lower extremity weakness, and absent reflexes. An extensive workup ruled out infectious and autoimmune causes, but a computed tomography angiogram (CTA) revealed severe cerebral vasoconstriction. Treatment included levetiracetam, intravenous magnesium, and nimodipine. The case highlights the challenge of differentiating RCVS from eclampsia in the postpartum period, emphasizing the importance of considering alternative diagnoses and brain CTA when RCVS is suspected, with calcium channel blockers potentially contributing to favorable neurological outcomes.

## Introduction

Eclampsia is a serious pregnancy complication known for causing seizures, which can be life-threatening. However, seizures occurring beyond 48 hours postpartum are less likely to be triggered by eclampsia, necessitating investigations for alternative causes. Reversible cerebral vasoconstriction syndrome (RCVS) in postpartum women, also known as Call-Fleming syndrome, is a rare condition that was first documented in 1988 and is characterized by transient constriction of cerebral blood vessels [1,2]. While the hallmark manifestation is typically a sudden-onset headache, RCVS can also present with a spectrum of neurological symptoms, including confusion, focal neurologic deficit, photophobia, phonophobia, and epileptic seizures. In postpartum women, RCVS tends to manifest within the first three months following childbirth [2,3]. This temporal association underscores the importance of considering RCVS in the differential diagnosis of postpartum patients presenting with abnormal neurological symptoms. The present case describes diagnostic and therapeutic challenges in an 18-year-old pregnant woman with eclampsia who developed abnormal neurological symptoms postpartum and was ultimately diagnosed with RCVS.

## Case presentation

An 18-year-old G1P0 Micronesian woman at 32 weeks of pregnancy, with a history of bilateral hearing loss treated with cochlear implants, presented with altered mental status, bilateral lower extremity weakness, and absent reflexes following an episode of eclampsia that necessitated emergent primary low transverse cesarean section (PLTCS). Eleven days earlier, she was admitted for sepsis secondary to pyelonephritis, treated with ceftriaxone, and discharged with oral cephalexin. Four days before the medicine consultation, she had a tonic-clonic seizure requiring PLTCS for non-reassuring fetal status. Post the operation, she was admitted to the intensive care unit and downgraded two days later, but she remained confused and had another seizure 48 hours postpartum. Medicine and neurology consultations were obtained, and levetiracetam and magnesium drip were initiated.

Upon examination, she was oriented to time, place, and person but was inattentive and easily distracted. Vital signs were unremarkable except for blood pressure of 155/119 mmHg. The patient appeared lethargic with a flat affect, and neurological examination revealed positive gaze-evoked nystagmus, 5/5 strength in bilateral upper extremities, 3/5 in bilateral lower extremities, intact light-touch sensations, positive cerebellar signs, and areflexia in patellar and Achilles reflexes. Plantar were flexor and there was no clonus.

Prior to admission, the patient was not taking any medications, supplements, or illicit drugs. The results of the main laboratory testing are summarized in Table 1. Lumbar puncture revealed WBC 1 cu/mm, red blood cell 0 cu/mm, protein 62 mg/dL, glucose 41 mg/dL, and no viral or bacterial organism was identified. It was negative for antinuclear antibody, venereal disease research laboratory (VDRL), oligoclonal bands, myelin basic protein, and autoimmune encephalitis panel (Table 2).

**Table 1:** Main laboratory data Hb: Hemoglobin, Hct: Hematocrit, Plt: Platelet, Na: Sodium, K: Potassium, Cl: Chloride, Mg: Magnesium, Ca: Calcium, BUN: Blood urea nitrogen, S.Cr: Serum creatinine, LDH: Lactate dehydrogenase, TP: Total protein, Alb: Albumin, AST: Aspartate aminotransferase, ALT: Alanine aminotransferase, ALP: Alkaline phosphatase, T-Bil: Total bilirubin, ANA: Anti-nuclear antibody, dsDNA: Double-stranded DNA, MOG: Myelin oligodendrocyte glycoprotein antibody, NMO: Neuromyelitis optica, AntiGQ1B: Ganglioside antibody

Complete blood count	Result	Reference range
WBC (10^3 ^/uL)	7.2	3.8-10.84
Hb (g/dl)	10.1	11.2-15.7
Hct (%)	30.6	34.1-44.9
Plt (10^3 ^/uL)	261	151-424
Biochemical findings		
Na (mEq/L)	137	133-145
K (mEq/L)	3.6	3.3-5.1
Cl (mEq/L)	98	95-108
Mg (mEq/L)	1.7	1.6-2.6
Ca (mEq/L)	8.5	8.3-10.5
Glucose (mg/dL)	80	70-99
BUN (mg/dL)	12	6.0-23
S.Cr (mg/dL)	0.7	0.6-1.4
TP (g/dL)	6.5	6.4-8.3
Alb (g/dL)	3.5	3.5-5.2
AST (IU/L)	20	<40
ALT (IU/L)	19	<42
ALP (IU/L)	120	35-129
T.Bil (mg/dL)	0.5	<1.2
LDH (IU/L)	238	135-250
Autoimmune workup findings		
ANA	<40	<40
Anti-dsDNA	<1	<4
Anti-MOG-IgG	Negative	Negative
NMO antibody	Negative	Negative
AntiGQ1B	Negative	Negative

**Table 2:** Cerebrospinal fluid analysis VDRL: Venereal disease research laboratory, AGNA: Antiglial nuclear antibody, AMPAR: Alpha-amino-3-hydroxy-5-methyl-4-isoxazole propionic acid receptor, Ab: Antibody, ANNA: Antineuronal nuclear antibody, CASPR2: Contactin-associated protein 2; CBA: Cell-binding assay/cell-based assay, CRMP-5: Collapsin response-mediator protein 5, DPPX: Dipeptidyl-aminopeptidase-like protein 6, GABA-BR: Gamma-aminobutyric acid receptor type B, GAD65: Glutamic acid decarboxylase 65-kd isoform, IFA: Indirect immunofluorescence assay, LGl1: Leucine-rich glioma-inactivated protein 1, mGluR1: Metabotropic glutamate receptor 1, NMDAR: N-methyl-D-aspartate receptor, PCCA: Purkinje cell cytoplasmic antibody

Cerebrospinal fluid analysis	Result	Reference range
Color	Colorless	Colorless
Appearance	Clear	Clear
RBC	0	0
WBC	1	<5
Neutrophils (%)	0	0
Lymphocytes (%)	100	<5
Monocytes (%)	0	0
Eosinophils (%)	0	0
Basophils (%)	0	0
Protein (mg/dl)	62	15-45
Sugar (mg/dl)	60	>60% of plasma value
Oligoclonal bands	Negative	Negative
Myelin basic protein	Negative	Negative
CSF meningitis/encephalitis panel		
*Escherichia coli* K1	Not detected	
Haemophilus Influenzae	Not detected	
Listeria mononocytogenes	Not detected	
Neisseria	Not detected	
Streptococcus agalactiae	Not detected	
Streptococcus pneumoniae	Not detected	
Cytomegalovirus	Not detected	
Enterovirus	Not detected	
Herpetic simplex virus type 1	Not detected	
Herpetic simplex virus type 2	Not detected	
Human herpes virus 6	Not detected	
Human parechovirus	Not detected	
Varicella	Not detected	
Cryptococcus	Not detected	
*Cryptococcus *antigen titer	Negative	
VDRL	Non-reactive	
Gram stain	Negative	
CSF encephalopathy/paraneoplastic panel		
AMPA-R Ab	Negative	
Amphiphysin	Negative	
AGNA-1	Negative	
ANNA-1	Negative	
ANNA-2	Negative	
ANNA-3	Negative	
CASPR2-IgG	Negative	
CRMP-5	Negative	
DPPX AB IFA	Negative	
GABA-B-R Ab	Negative	
GAD65 Ab nmol/L	Negative	
IgLON5 IFA	Negative	
LGI1-IgG	Negative	
mGLuR1 Ab	Negative	
NMDAR Ab	Negative	
PCCA-1	Negative	
PCCA-Tr	Negative	

**Figure 1:**
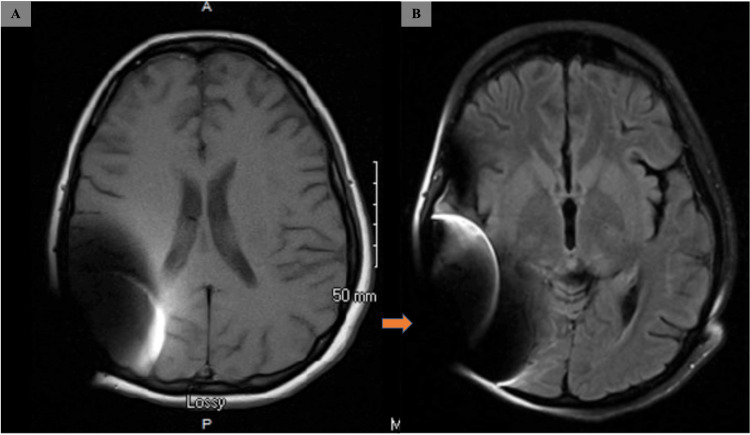
Brain MRI of the patient A: No acute intracranial abnormalities observed on MRI of the brain without contrast; B: Right cerebellum markedly limited secondary to artifact from a cochlear implant (orange arrow)

**Figure 2:**
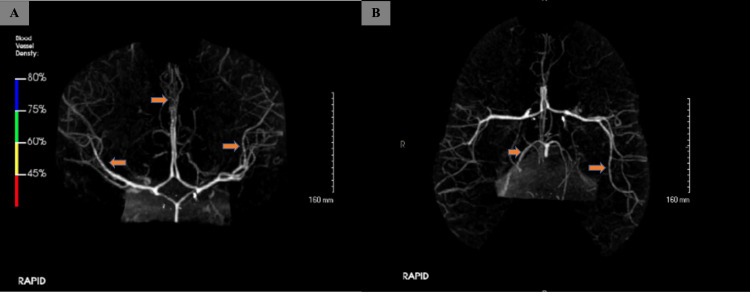
CTA of the brain A: Mild to moderate diffuse narrowing of the ACAs and MCAs (orange arrows); B: Mild to moderate diffuse narrowing of MCAs and PCAs (orange arrows) CTA: Computed tomography angiography, ACAs: Anterior cerebral arteries, MCAs: Middle cerebral arteries, PCAs: Posterior cerebral arteries

Initially, Miller-Fischer syndrome was a main differential diagnosis; however, it could not explain the patient's altered mental status and recurrent seizures. Additionally, a 16-hour EEG showed nonspecific mild diffuse disturbance of cerebral function. Brain MRI was normal except for artifact due to her cochlear implant (Figure 1), but computed tomography angiogram (CTA) of the brain showed diffuse severe narrowing of the anterior and posterior circulation, which led to the diagnosis of RCVS (Figure 2).

Given the persistent encephalopathy due to RCVS, the patient was transferred to the neuroscience ICU (NSICU) for close monitoring of her symptoms and intensive medical therapy, including anti-seizure medications and nimodipine as a vasodilatory agent. Fortunately, serial CTA brain showed improving vasospasm, and daily transcranial Doppler revealed improving velocity. The patient was discharged on hospital day 29 with two weeks of nimodipine and levetiracetam 500 mg twice daily.

## Discussion

The present report describes a challenging case of RCVS in the setting of a patient with known eclampsia. While clinicians tend to associate peripartum seizures with eclampsia as in this case, persistent seizures beyond 48 hours postpartum require an alternate diagnosis such as intracerebral hemorrhage, subarachnoid hemorrhage, encephalitis, encephalopathy, CNS vasculitis, epilepsy, or postpartum angiopathy [2]. In particular, RCVS is a rare yet reversible cause of peripartum seizures with encephalopathy. It manifests as reversible cerebral vasoconstriction, typically occurring within the initial three weeks postpartum [3]. Reversible cerebral vasoconstriction syndrome has been linked to various factors, including pregnancy, migraines, vasoconstrictive drugs, aneurysms, and neurosurgical procedures. Diagnostic imaging with CTA reveals a widespread reduction in the caliber of cerebral vessels and their branches.

The optimal treatment for RCVS remains uncertain, but existing studies recommend calcium channel blockers such as verapamil or nimodipine. Additionally, intravenous magnesium therapy is suggested for its vasodilatory effects [4-6]. The study by Marsh et al. indicates a potential superiority of verapamil among calcium channel blockers [6]; however, the effectiveness of nimodipine in addressing vasoconstriction has been observed in McIlroy et al.'s study [7]. Further randomized controlled trials are required to establish a definitive treatment approach. Previous studies report a good prognosis with self-limited symptoms and complete recovery within three months [8]. Recurring episodes of RCVS in future pregnancies are uncommon [9].

Early diagnosis and treatment are essential to reduce the risk of stroke associated with RCVS [10]. However, as in the present case, symptoms of RCVS are nonspecific, and patients tend to be prematurely diagnosed and treated as a more common syndrome, such as eclampsia. It is essential to thoroughly assess the situation and consider other possible explanations to avoid premature closure.

## Conclusions

Reversible cerebral vasoconstriction syndrome encompasses a spectrum of disorders marked by the transient constriction of cerebral arteries, leading to multifocal narrowing. Despite the generally favorable clinical prognosis associated with RCVS, it is crucial to acknowledge the potential for severe complications, including major strokes that can result in significant disability or even mortality in some cases. Recognition of RCVS as a potential diagnosis becomes particularly imperative in patients presenting with prolonged altered mental status, and its consideration is paramount in specific clinical scenarios, such as pregnancy. In addition, the optimal treatment for RCVS is unclear but existing studies recommended calcium channel blockers such as nimodipine or verapamil. The multifaceted nature of RCVS underscores the importance of a comprehensive medical history, including the identification of potential triggers or predisposing factors, for a more accurate and timely diagnosis. Clinicians should remain vigilant to the manifestations of RCVS, as early intervention and management can significantly impact the overall clinical outcome.
